# Identifying Imaging Genetics Biomarkers of Alzheimer’s Disease by Multi-Task Sparse Canonical Correlation Analysis and Regression

**DOI:** 10.3389/fgene.2021.706986

**Published:** 2021-08-05

**Authors:** Fengchun Ke, Wei Kong, Shuaiqun Wang

**Affiliations:** College of Information Engineering, Shanghai Maritime University, Shanghai, China

**Keywords:** imaging genetics, sparse canonical correlation analysis, magnetic resonance imaging, positron emission tomography, single nucleotide polymorphisms, multi-task learning

## Abstract

Imaging genetics combines neuroimaging and genetics to assess the relationships between genetic variants and changes in brain structure and metabolism. Sparse canonical correlation analysis (SCCA) models are well-known tools for identifying meaningful biomarkers in imaging genetics. However, most SCCA models incorporate only diagnostic status information, which poses challenges for finding disease-specific biomarkers. In this study, we proposed a multi-task sparse canonical correlation analysis and regression (MT-SCCAR) model to reveal disease-specific associations between single nucleotide polymorphisms and quantitative traits derived from multi-modal neuroimaging data in the Alzheimer’s Disease Neuroimaging Initiative (ADNI) cohort. MT-SCCAR uses complementary information carried by multiple-perspective cognitive scores and encourages group sparsity on genetic variants. In contrast with two other multi-modal SCCA models, MT-SCCAR embedded more accurate neuropsychological assessment information through linear regression and enhanced the correlation coefficients, leading to increased identification of high-risk brain regions. Furthermore, MT-SCCAR identified primary genetic risk factors for Alzheimer’s disease (AD), including rs429358, and found some association patterns between genetic variants and brain regions. Thus, MT-SCCAR contributes to deciphering genetic risk factors of brain structural and metabolic changes by identifying potential risk biomarkers.

## Introduction

Imaging genetics has recently emerged as a method for investigating imaging and genetic biomarkers related to diseases such as Alzheimer’s disease (AD) (Bogdan et al., 2017). Identified neuroimaging and genetics biomarkers can provide a complementary understanding of the brain’s structure and metabolism (Zhang et al., 2011). Moreover, the vast amounts of diagnostic and neuropsychological information from various perspectives enable the discovery of disease-specific biomarkers. Therefore, it is essential to simultaneously analyze multiple neuroimaging techniques, such as magnetic resonance imaging (MRI), fluorodeoxyglucose positron emission tomography (FDG-PET), genotyping, and clinical diagnostic data. In this study, we aimed to build a model to identify disease-specific biomarkers across multiple imaging modalities, which can be used as an effective clue for disease diagnosis and targeted therapy.

Numerous studies have attempted to identify the associations between genotypic data such as single nucleotide polymorphisms (SNPs) and neuroimaging quantitative traits (QTs) (Rasetti and Weinberger, 2011). Because genotypic data and imaging QTs are multivariate, several bi-multivariate methods have been proposed to better characterize their associations. Liu et al. explored parallel independent component analysis (PICA) to detect the associations between brain function and genetic variants. However, this method cannot restore meaningful SNPs and regions of interest (ROIs), which has led to a lack of reasonable biomarker interpretation (Liu et al., 2009). Sparse canonical correlation analysis (SCCA) has a strong capability for bi-multivariate association identification and interpretable variable selection. Accordingly, many efforts have attempted to apply SCCA to neuroimaging genetics. Boutte et al. introduced an SCCA model with least absolute shrinkage and selection operator (LASSO) constraints on neuroimaging genetics data fusion (Boutte and Liu, 2010). Hao et al. presented a multi-view SCCA model to establish associations between SNPs, QTs, and cognitive outcomes (Hao et al., 2017). However, these multi-view SCCA models are a simple extension to conventional SCCA models. The requirement that SNP canonical weight vectors associate with all modal data is too strict, and could result in not making full use of all modal information. To address this limitation, Du et al. developed a multi-task SCCA model that could be used to jointly analyze SNPs and multiple neuroimaging data by treating each association as an individual learning task (Du et al., 2021). However, this model’s neglect of diagnostic information means that biomarkers identified by these multiple-data models may not be sufficiently disease-specific.

To detect more complex and meaningful associations, studies to date have applied diagnostic information into SCCA methods (Yan et al., 2018; Du et al., 2020). Yan et al. proposed an outcome-relevant SCCA model based on a subject similarity matrix (Yan et al., 2018). Du et al. integrated multi-task SCCA and logistic regression in a sophisticated model to identify robust disease-related imaging and genetic patterns by incorporating diagnostic status information (Du et al., 2020). Classified diagnostic information, such as AD, mild cognitive impairment (MCI), and healthy control (HC), facilitates the association between SNPs and QTs; however, roughly dividing the disease stages does not provide any more accurate information than do continuous neuropsychological assessments measured from different angles.

To address the above problems, we proposed a novel SCCA model with the capacity to extract disease-specific biomarkers across multiple neuroimaging modalities. The proposed multi-task sparse canonical correlation analysis and regression (MT-SCCAR) model integrates multi-task SCCA and multi-task linear regression in a fused model and uses multiple cognitive scores (CSs) as auxiliary information to induce associations between SNPs and QTs. Multi-task sparse canonical correlation analysis and regression considers the relationships within subjects from different disease courses and can find disease-specific biomarkers. We also considered underlying hierarchical information among SNPs by modeling structural relationships as divided by gene or by linkage disequilibrium (LD) in a group sparsity penalty. To evaluate MT-SCCAR’s effectiveness, we performed extensive experiments to find associations between SNPs and two imaging QTs, including gray matter density and standard uptake value ratio (SUVR) extracted from MRI and positron emission tomography (PET), respectively. Compared with the other two multi-modal SCCA models that used real Alzheimer’s Disease Neuroimaging Initiative (ADNI) cohort data, MT-SCCAR not only outperformed these models in its ability to identify genetic AD risk factors, but also detected robust AD brain risk regions across multiple neuroimaging modalities. Thus, our proposed model has the potential to understand disease mechanisms from both structural and metabolic perspectives.

## Materials and Methods

### Data Sources and Preprocessing

Real neuroimaging and genetic data used in this study were obtained from the ADNI1 database. A total of 305 non-Hispanic Caucasian subjects with genotype, neuroimaging, and cognitive assessment data at the ADNI1 baseline were downloaded from the LONI website,^1^ including 83 HC, 148 MCI, and 74 AD subjects. The Mini-Mental State Examination (MMSE) is a numeric scale to test cognitive functions, including attention, calculation, and responsiveness to simple commands (Tombaugh and McIntyre, 1992). The Functional Activities Questionnaire (FAQ) evaluates instrumental activities of daily life, such as financial management and meal preparation (Teng et al., 2010). The Alzheimer’s Disease Assessment Scale Cognitive Subscale (ADAS-Cog) mainly measures cognitive ability such as word recall, comprehension of spoken language, and orientation (Cano et al., 2010). Table 1 shows the characteristics of the subjects.

**TABLE 1:** Characteristics of the subjects.

Subjects	HC	MCI	AD
Number	83	148	74
Gender(M/F)	50/33	98/50	39/35
Age(mean ± std)	77.76 ± 4.59	76.62 ± 6.92	76.96 ± 6.91
Education(mean ± std)	15.68 ± 3.09	15.88 ± 2.77	14.27 ± 3.37
MMSE (mean ± std)	29.18 ± 1.11	26.09 ± 3.14	20.57 ± 3.20
FAQ (mean ± std)	0.54 ± 1.25	6.62 ± 8.96	19.75 ± 3.50
ADAS-Cog(mean ± std)	6.00 ± 2.89	13.72 ± 3.03	26.09 ± 11.64

#### Genotyping Data and Processing

Genotypes for 305 subjects were performed using the Illumina HumanHap610-Quad BeadChips from the ADNI1 database. The SNP data were lifted to hg19 build using lift over tool (Kent et al., 2002). To get pure SNP data, we used a genetic analysis tool PLINK (Purcell et al., 2007) to filter the SNPs using the following quality control criteria: gender check, sibling pair identification, call rate check (<90%) per subject and SNP marker, the Hardy-Weinberg Equilibrium (HWE *p* < 10–6), and marker removal by the minor allele frequency (MAF < 0.05), SNP data were further imputed using Michigan imputation server to estimate the missing genotypes based on the HRC r1.1 2016 panel (Das et al., 2016). The post-imputation quality control used the rsq > 0.3 and MAF of 0.1 (Li et al., 2010).

**FIGURE 1:**
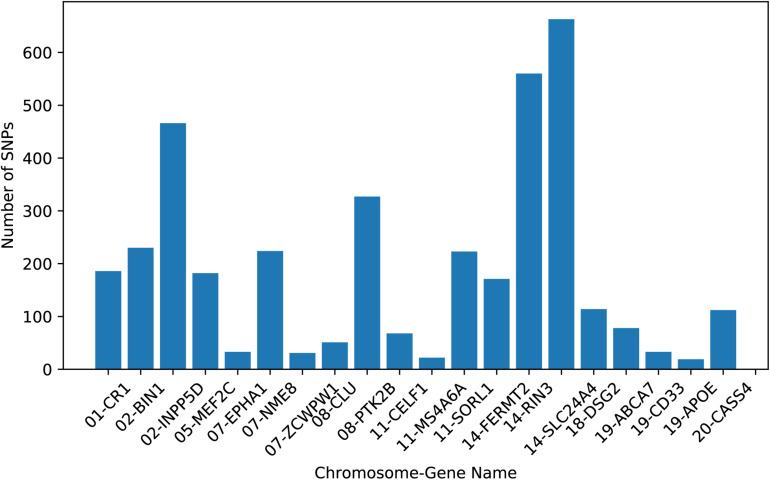
The numbers of SNPs belonging to each AD risk gene used in this study.

Since our study focused on the top 20 AD risk genes listed on the AlzGene database^2^ and references (Tanzi et al., 2007; Wang et al., 2012a). After imputation, we selected all the SNPs within ± 5k base pairs of the gene boundary using the ANNOVAR annotation (Wang et al., 2010). The above procedures yielded 3793 SNPs belonging to the top 20 risk genes. Figure 1 presents the AD risk genes and the number of pre-selected SNPs. Moreover, considering the structural relationship among SNPs, we used Haploview (Barrett et al., 2004) to divide the LD block using the LD-Spline algorithm with *D*′ > 0.8, resulting in 209 blocks containing 3770 SNPs. A total of 894 tag SNPs were also assigned by Haploview in pairwise mode and an *r*^2^ threshold was set to 0.8. These tagged SNPs represented the genetic variation across a particular region and could facilitate the association study (Montpetit et al., 2006). Furthermore, each SNP value was coded in an additive fashion to reflect the number of minor alleles.

#### Neuroimaging Data and Processing

The baseline 1.5T MRI scans were aligned to the standard Montreal Neurological Institute (MNI) space, resampled to 2 × 2 × 2 mm^3^ voxels, registered by SPM software package (Ashburner and Friston, 2007). Then, we extracted the gray matter tissue from the MRI scans and calculated mean gray matter densities of 116 ROIs based on MarsBar AAL atlas (Tzourio-Mazoyer et al., 2002). After removing 26 ROIs of the cerebellum, mean gray matter densities of 90 ROIs were used as QTs in our study.

The FDG-PET scans were co-registered to each subject’s same visit MRI scans and normalized to MNI space by SPM tool. We further excluded white matter regions by masking the PET with gray matter masks obtained by the segmentation of the same subject’s co-registered MRI. Then, the PET scans were normalized into the cerebellar gray matter reference region defined on the AAL atlas to generate SUVR images. After this, we used SUVR of 90 ROIs as QTs in our study by removing the 26 ROIs of cerebellum. Moreover, all the QTs were adjusted to exclude the influence of gender, age, and education.

### Methods

In this paper, we denote lowercase letters as vectors, uppercase letters as matrices. ||**x**||_2_ denotes the Euclidean norm, ||**X**||_2,1_ denotes the sum of the Euclidean norms of the rows of **X**, and ||**X**||_1,1_ denotes the absolute sum of all elements of *X*.

#### The CS-Related Features Selection Model for Imaging Genetics

Assuming that there are _*n*_ subjects with *p*SNPs, *q*ROIs from *M* imaging modalities, and *G* different cognitive outcomes. We used **X** ∈ *R*^*n*×*p*^,**Y**_*m*_ ∈ *R*^*n*×*q*^(*m*=1,…,*M*), and **z**_*g*_ ∈ *R*^*n*×1^(*g*=1,…,*G*) to represent genetic data, multiple imaging data, and cognitive scores, respectively. The basic principle of MT-SCCAR is to find **U** ∈ *R*^*p*×*M*^ and **V** ∈ *R*^*q*×*M*^ to maximize the correlation between **Xu**_*m*_ and **Y**_*m*_**u**_*m*_, where*u*_*im*_ indicates the weight of the *i*th SNP for the *m*th modality, and*v*_*jm*_ indicates the weight of the *j*th ROI for the *m*th modality. To identify imaging genetic biomarkers that are relevant to CS and disease, the multi-task linear regression objective was combined with the multi-task SCCA (MTSCCA) objective, which can be formulated as:

(1) $$\min_{U,V}{ℒ}_{R}\,\left(V\right)+{ℒ}_{S\,C\,C\,A}\,\left(U,V\right)+\Omega \,\left(U\right)+\Omega \,\left(V\right).$$

**FIGURE 2:**
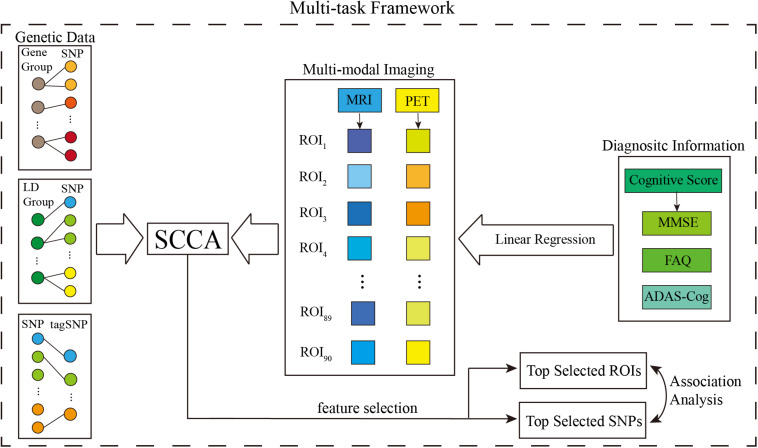
Schematic illustration of MT-SCCAR.

The above model consists of four parts,ℒ_*R*_(**V**) detects disease-relevant imaging QTs. ℒ_*SCCA*_(**U**,**V**) captures the bi-multivariate associations between SNPs and multiple imaging QTs. Ω(**U**) and Ω(**V**) are the regularization terms to enforce sparsity of **U** and **V**, so only a small number of interpretable variables can be selected. This model integrates the advantages of MTSCCA and linear regression, which has a certain superiority in using complementary cognitive information. Figure 2 provides a schematic overview of MT-SCCAR. SNPs were classified into the same group by either gene or LD. Accordingly, SNPs with gene or LD information and tagSNPs were input to the SCCA component separately, which was used to establish the relationships between genetic data and multiple imaging data. The linear regression component was used to introduce CSs into the SCCA part. The multi-task modeling method guaranteed the ability to process multiple imaging and CS data. Unlike conventional unsupervised SCCA models, MT-SCCAR is a supervised SCCA model, which considers the relationships within subjects from different disease courses.

#### The Linear Regression Model for CS-QT Associations

In the proposed model, the associations between CSs and multi-modal neuroimaging QTs were established by multi-task regression. For each task, we built a regression model for revealing CS-related neuroimaging QTs:

(2) $${ℒ}_{R}\,\left(V\right)=\sum_{m=1}^{M}\sum_{c=1}^{C}\sum_{l=1}^{n}{||{\text{v}}_{m}^{\text{T}}\,{\text{y}}_{m}^{l}-z_{c}^{l}||}_{2}^{2},$$

where *M* is the number of neuroimaging modalities, *C* is the number of cognitive assessments, and *n* is the total amount of subjects. **v**_*m*_ is the canonical weight of QTs for the *m*th modalities,${\text{y}}_{m}^{l}$ is the neuroimaging data vector of the *l*th subjects for the *m*th modalities, and *z^l*_*c*_ is the score of the *l*th subjects for the *c*th cognitive assessments. This multi-task regression model can jointly utilize neuropsychological assessments from different complementary perspectives.

#### The MTSCCA Model for SNP-QT Associations

Unlike conventional multi-view SCCA models, MTSCCA learns multiple SCCA tasks together by treating each imaging modality association model as a task. This model was proposed by Du et al. (2021) and can be defined as:

(3) $$\min_{u_{m},v_{m}}\sum_{m=1}^{M}-{\text{u}}_{m}^{\text{T}}\,{\text{X}}^{\text{T}}\,{\text{Y}}_{m}\,{\text{v}}_{m}\,s.t.{||{\text{Xu}}_{m}||}_{2}^{2}=1,{||{\text{Y}}_{m}\,{\text{v}}_{m}||}_{2}^{2}=1,\forall m.$$

For canonical weights **U** and **V**, each column **u**_*m*_ and **v**_*m*_ represents an individual learning task for different modalities. The main advantage of this multi-task strategy is that SNP canonical weight vectors do not need to be associated with all imaging modalities simultaneously. Each task focuses on identifying SNPs that are associated with only one imaging modality.

#### The Regularization Terms

Multiple neuroimaging modalities can provide more comprehensive information in terms of both structural and functional perspectives. In our model, two principal tasks corresponded to two neuroimaging modalities. MT-SCCAR should be able to identify neuroimaging QTs shared among multiple modalities and to enforce individual level sparsity. Hence, Ω(**V**) was composed of two parts, which can be defined as:

(4) $$\Omega \,\left(V\right)=\lambda_{v\,1}\,{||V||}_{2,1}+\lambda_{v\,2}\,{||V||}_{1,1},$$

where λ_*v*1_ and λ_*v*2_ are positive parameters and can be tuned via cross-validation.

The first penalty was defined as:

(5) $${||V||}_{2,1}=\sum_{i=1}^{q}\sqrt{\sum_{m=1}^{M}{\text{V}}_{i,j}^{2}}=\sum_{i=1}^{q}{||{\text{V}}_{i,:}||}_{2},$$

This term aims to enforce task-consistent (modality-consistent) sparsity on **V**, which encourages multi-modal imaging QTs to share similar canonical weights.

The second penalty was defined as:

(6) $${||V||}_{1,1}=\sum_{j=1}^{q}\sum_{m=1}^{M}\left|v_{j\,m}\right|,$$

This term indicates the absolute sum of all elements of **V**, which helps to screen the entire ROIs to find the relevant ROIs.

Similarly, the regularization terms of **U** also include the above two penalties, which can help discover SNPs that may affect multiple brain regions. It is common knowledge that some SNPs located in the same gene or LD block often have similar functions and are jointly related to specific ROIs. It is essential to model underlying hierarchical information among SNPs by adding an extra penalty. Therefore, we defined Ω(**U**) as follows:

(7) $$\Omega \,\left(U\right)=\lambda_{u\,1}\,{||U||}_{2,1}+\lambda_{u\,2}\,{||U||}_{1,1}+\lambda_{u\,3}\,{||U||}_{G},$$

where λ_*u*1_, λ_*u*2_, and λ_*u*3_ are positive parameters, the third penalty (Wang et al., 2012a) can be formulated as:

(8) $${||U||}_{G}=\sum_{k=1}^{K}\sqrt{\sum_{i\in g_{k}}\sum_{j=1}^{M}u_{i\,j}^{2}},$$

where *K* denotes the number of groups divided by gene or LD. This penalty penalizes canonical weights as a whole for each task and thus can fully use the structural information.

#### The Optimization Algorithm

In order to address the problem defined in Equation (1), according to the method that has been well studied previously (Du et al., 2021), we can rewrite Equation (1):

$$\min_{U,V}\sum_{m=1}^{M}\sum_{c=1}^{C}\sum_{l=1}^{n}{||{\text{v}}_{m}^{\text{T}}\,{\text{y}}_{m}^{l}-z_{c}^{l}||}_{2}^{2}+\sum_{m=1}^{M}{||{\text{Xu}}_{m}-{\text{Y}}_{m}\,{\text{v}}_{m}||}_{2}^{2}+$$

(9) $$\lambda_{v\,1}\,{||V||}_{2,1}+\lambda_{v\,2}\,{||V||}_{1,1}+\lambda_{u\,1}\,{||U||}_{2,1}+\lambda_{u\,2}\,{||U||}_{1,1}+\lambda_{u\,3}\,{||U||}_{G}\,s.t.{||{\text{Xu}}_{m}||}_{2}^{2}=1,{||{\text{Yv}}_{m}||}_{2}^{2}=1,\forall m.$$

We then use the Lagrange multiplier to solve this problem by taking the partial derivatives of Equation (9) regarding **u**_*m*_ and**v**_*m*_ separately, which can change the formula from non-convex to convex.

First, we treat **U** as constant, the Lagrange multiplier of Equation (9) can be simplified as:

$$\sum_{m=1}^{M}\sum_{c=1}^{C}\sum_{l=1}^{n}{||{\text{v}}_{m}^{\text{T}}\,{\text{y}}_{m}^{l}-z_{c}^{l}||}_{2}^{2}+\sum_{m=1}^{M}{||{\text{Xu}}_{m}-{\text{Y}}_{m}\,{\text{v}}_{m}||}_{2}^{2}+$$

(10) $$\lambda_{v\,1}\,{||V||}_{2,1}+\lambda_{v\,2}\,{||V||}_{1,1}+\gamma_{v}\,\sum_{m=1}^{M}{||{\text{Y}}_{m}\,{\text{v}}_{m}||}_{2}^{2}$$

by dropping the constant terms, and γ_*v*_ is a positive parameter. For each **v**_*m*_, We further take the partial derivatives of Equation (10) and let the result be zero:

$${\text{Y}}_{m}^{\text{T}}\,{\text{Y}}_{m}\,{\text{v}}_{m}-\sum_{c=1}^{C}{\text{Y}}_{m}^{\text{T}}\,{\text{z}}_{c}-{\text{Y}}_{m}^{\text{T}}\,{\text{Xu}}_{m}+\lambda_{v\,1}\,{\text{D}}_{v\,1}\,{\text{v}}_{m}+\lambda_{v\,2}\,{\text{D}}_{v\,2}\,{\text{v}}_{m}$$

(11) $$+\left(\gamma_{v}+1\right)\,{\text{Y}}_{m}^{\text{T}}\,{\text{Y}}_{m}\,{\text{v}}_{m}=0,$$

where **D**_*v*1_ is a diagonal matrix with the *i*th element as $\frac{1}{2\,{||v_{i,:}||}_{2}}\left(i\in \left[1,q\right]\right)$, and **D**_*v*2_ is a diagonal matrix with *i*th element as $\frac{1}{2\,{||v_{i\,m}||}_{2}}\left(i\in \left[1,q\right],\text{and}m\in \left[1,M\right]\right)$. Obviously, we can take an iterative rule to solve this problem since both **D**_*v*1_ and **D**_*v*2_ are rely on canonical weights **V**. This rule can be formulated as:

(12) $${\text{v}}_{m}={\left({\text{Y}}_{m}^{\text{T}}\,{\text{Y}}_{m}+\lambda_{v\,1}\,{\text{D}}_{v\,1}+\lambda_{v\,2}\,{\text{D}}_{v\,2}+\left(\gamma_{v}+1\right)\,{\text{Y}}_{m}^{\text{T}}\,{\text{Y}}_{m}\right)}^{-1}\left(\sum_{c=1}^{C}{\text{Y}}_{m}^{\text{T}}\,{\text{z}}_{c}+{\text{Y}}_{m}^{\text{T}}\,{\text{Xu}}_{m}\right).$$

Then, we treat **V** as a constant, the Lagrange multiplier of Equation (9) can be simplified as:

(13) $$\sum_{m=1}^{M}{||{\text{Xu}}_{m}-{\text{Y}}_{m}\,{\text{v}}_{m}||}_{2}^{2}+\lambda_{u\,1}\,{||U||}_{2,1}+\lambda_{u\,2}\,{||U||}_{1,1}+\lambda_{u\,3}\,{||U||}_{G}+\gamma_{u}\,{||\mathrm{Xu}||}_{2}^{2}$$

by dropping the constant terms, and γ_*u*_ is also a positive parameter. Similar to **v**_*m*_, for **U**, we let the partial derivatives of Equation (13) to be zero:

(14) $$-{\text{X}}^{\text{T}}\,𝕐+\lambda_{u\,1}\,{\text{D}}_{u\,1}\,\text{U}+\lambda_{u\,2}\,{\text{D}}_{u\,2}\,\text{U}+\lambda_{u\,3}\,{\text{D}}_{u\,3}\,\text{U}+\gamma_{u}\,{\text{X}}^{\text{T}}\,\text{X}\,U=0,$$

where **D**_*u*1_ is a diagonal matrix with the *i*th element as $\frac{1}{2\,{||u_{i,:}||}_{2}}\left(i\in \left[1,p\right]\right)$, **D**_*u*2_ is a diagonal matrix with *i*th element as $\frac{1}{2\,{||u_{i\,m}||}_{2}}\left(i\in \left[1,p\right],\text{and}m\in \left[1,M\right]\right)$, **D**_*u*3_ is a block diagonal matrix with element as $\frac{1}{2\,{||U_{k,:}||}_{\text{F}}}{\text{I}}_{k}\left(k\in \left[1,K\right]\right)$, *I*_*k*_ is an identity matrix of the same size with *k*th SNP groups, and *𝕐*=[**Y**_1_**v**_1_**Y**_2_**v**_2_…**Y**_*m*_**v**_*m*_]. Hence, the iterative rules can be formulated as:

(15) $$U={\left(\lambda_{u\,1}\,{\text{D}}_{u\,1}+\lambda_{u\,2}\,{\text{D}}_{u\,2}+\lambda_{u\,3}\,{\text{D}}_{u\,3}+\left(\gamma_{u}+1\right)\,{\text{X}}^{\text{T}}\,\text{X}\right)}^{-1}\,{\text{X}}^{\text{T}}\,𝕐.$$

Based on the above analysis, the optimization algorithm of the proposed method is shown in Table 2. We can update **V** and **U** alternatively in each iteration until the predefined convergence criterion is satisfied.

**TABLE 2:** Specific procedure of MT-SCCAR algorithm.

Algorithm: MT-SCCAR algorithm
Input: The genetic data **X** ∈ *R*^*n*×*p*^, the neuroimaging data **Y** ∈ *R*^*n*×*q*^ of *M* modalities, and the CS data **Z** ∈ *R*^*n*×*C*^. λ_*u*1_,λ_*u*2_,λ_*u*3_,λ_*v*1_,λ_*v*2_,γ_*u*_,andγ_*v*_.
Ensure: canonical weights **V**and **U**
1: While not converged regarding to**V**, **U** do
2:Update the diagonal matrix **D**_*v*1_ and **D**_*v*2_;
3:Solve **v**_*m*_according to Equation (12);
4:Normalize **v**_*m*_ so that ${||{\text{Yv}}_{m}||}_{2}^{2}=1$;
5:Update the diagonal matrix **D**_*u*1_, **D**_*u*2_ and **D**_*u*3_;
6:Solve **U** according to Equation (15);
7:Normalize **u**_*m*_ so that ${||{\text{Xu}}_{m}||}_{2}^{2}=1$;
8: End while

## Results and Discussion

### Experimental Settings

To comprehensively evaluate the effectiveness of our proposed MT-SCCAR model, two similar models that can analyze multi-modal data were compared with MT-SCCAR. They are three-view SCCA (TSCCA) and MTSCCA. Three-view SCCA can process neuroimaging, genetics, and cognitive scores data by extending conventional two-view association to three data types. MTSCCA was used to evaluate the regression part of our proposed model performance.

There are seven parameters in our model. Tuning all these parameters will pay a high cost. In our experiment, we fixed γ_*u*_ and γ_*v*_ to 1 since they mainly control the amplitude of **V** and **U** (Chen and Liu, 2011). To tune these parameters to appropriate values, we adopted a nested five-fold cross-validation strategy. Specifically, we tuned them in the range of {10^−3^,10^−2^,10^−1^,1, 10,10^2^, 10^3^} until the highest mean testing canonical correlation coefficients (CCCs) was generated in the inner loop. CCC was defined as the Pearson correlation coefficient between **X***u* and **Y***v*, and can be used as a quantitative measure of SCCA model performance (Hao et al., 2017). For multi-task learning, CCC can be calculated by *corr*(**X**_*m*_**u**_*m*_,**Y**_*m*_**v**_*m*_) for *m*th task. Also, we terminated the iteration when both $\text{max}\,\left|u_{i}^{\left(t+1\right)}-u_{i}^{t}\right|\leq 10^{-5}$ and $\text{max}\,\left|v_{j}^{\left(t+1\right)}-v_{j}^{t}\right|\leq 10^{-5}$ were satisfied. All models in our experiment have taken the same parameter adjustment steps.

### Results on Synthetic Data

**FIGURE 3:**
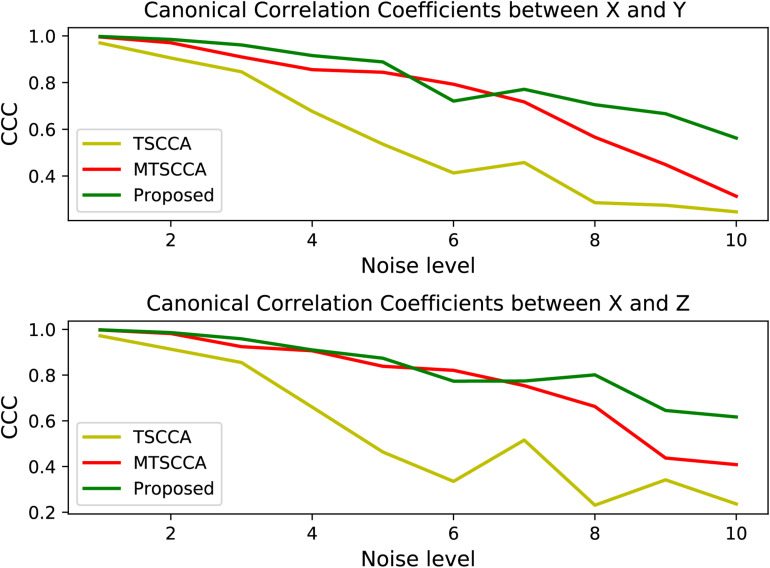
Comparison of CCCs under various noise levels for three models.

**FIGURE 4:**
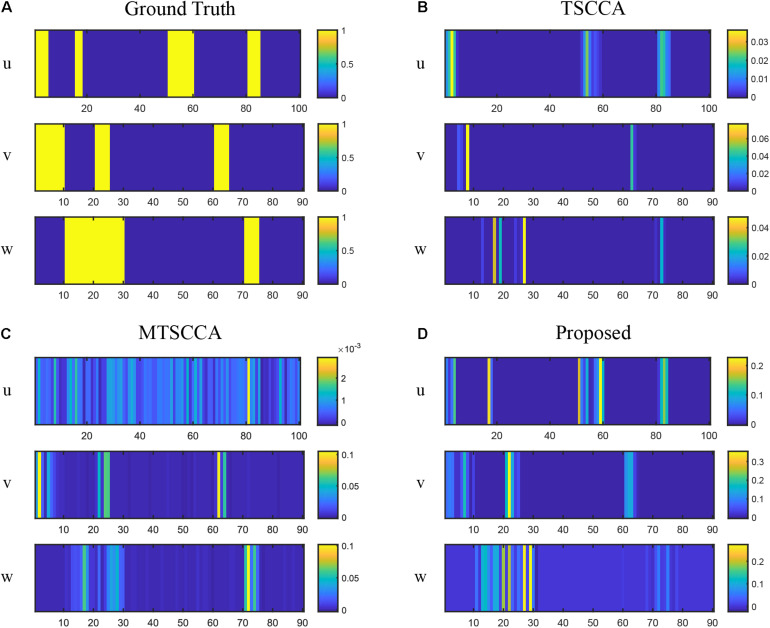
Comparison of canonical weights on synthetic data with the high noise level. **(A)** The ground truth canonical weights. **(B)** The estimated canonical weights of TSCCA. **(C)** The estimated canonical weights of MTSCCA. **(D)** The estimated canonical weights of the proposed model.

We generated ten synthetic datasets with the same ground truth of loading vectors but different noise levels. Assuming that **X** ∈ *R*^*n*×*p*^,**Y** ∈ *R*^*n*×*q*^, and **Z** ∈ *R*^*n*×*q*^ denote SNP, MRI, and PET for all synthetic data sets, respectively. **X** was generated by **X**=**ul**+**e**,**Y** was generated by **Y**=**vl**+**e**, and **Z** was generated by **Z**=**w***l*+**e**, where **u**, **v**, and **w**are known loading vectors, **l** is a latent vector with a 3-component Gaussian distribution to simulate the disease course (Yan et al., 2018), and **e** is derived from the Gaussian distribution $N\,\left(0,\sigma_{e}^{2}\right)$ with $\sigma_{e}^{2}$ as the noise variance. In our study, *n*, *p*, and *q* were set to 90, 100, and 90, respectively. All the 90 samples were classed into three groups with centers -5, 0, 5. For neuropsychological assessment data, **c** was generated by **c**=**l**+**e**. To assess the model performance at various noise levels, we tested different noise variances ranging from 1 to 10, with a step size of 1. The five-fold cross-validation results are shown in Figures 3, 4.

Figure 3 plots the testing CCC for three models with changing noise levels. Higher CCC indicates better performance in identifying underlying associations. As expected, the performance decreased with increased noise levels for all models. All three models performed similarly well at low noise levels. Models with the multi-task framework (MTSCCA, MT-SCCAR) performed better than TSCCA at medium noise levels. Then MT-SCCAR outperformed the other two models as the noise level was further increased, suggesting that MT-SCCAR had a strong ability to resist noise. Figure 4 shows the true signal of canonical weights and canonical weights estimated by three models with a noise level of 10. Important features were highlighted in the heatmaps displaying ground truth. We could clearly observe that the weight **u** estimated by MTSCCA was ambiguous. It was therefore difficult to recognize important features. TSCCA did not identify complete important features. MT-SCCAR estimated the best canonical weights that were consistent with the ground truths. These results implied that the proposed model had the potential to extract important features in real neuroimaging genetics studies.

### Results on Neuroimaging and Genetics Data

In real neuroimaging genetics data application, all subjects with SNP, MRI, PET, and three different cognitive information data were inputted into MT-SCCAR.A total of 3793 SNPs with LD or gene group information and 894 tag SNPs were used separately. The group sparsity penalty treated each tagSNP as an individual group. We then averaged the CCCs based on five-fold cross-validation, representing the mean strength of identified associations between SNPs and two imaging QTs.

As illustrated in Table 3, TSCCA achieved the highest training CCCs but performed poorly in testing CCCs. These unreasonable results may be caused by overfitting (Du et al., 2021). Multi-task sparse canonical correlation analysis and regression achieved the highest testing CCCs on both MRI and PET. Specifically, MT-SCCAR (LD) and MT-SCCAR (gene) achieved the highest testing CCC on SNP-MRI association and SNP-PET association, respectively. Notably, MT-SCCAR (gene) achieved relatively small testing CCC on SNP-MRI association; MT-SCCAR (LD) achieved a more balanced result than those of MT-SCCAR (gene), which indicates that using LD group information is more beneficial than using gene group information. The training CCCs of MT-SCCAR with tagSNP were higher than those of MT-SCCAR with group information since the different numbers of SNPs were used. Moreover, MTSCCA also performed better than TSCCA, which means the superiority of multi-task models when dealing with multiple imaging QTs and genetic data.

**TABLE 3:** Comparison of canonical correlation coefficients (mean ± std) in terms of each model.

	Training CCCs		Testing CCCs	
	SNP-MRI	SNP-PET	SNP-MRI	SNP-PET
TSCCA	**0.82 ± 0.01**	**0.82 ± 0.01**	0.21 ± 0.05	0.23 ± 0.03
MTSCCA	0.55 ± 0.05	0.46 ± 0.11	0.21 ± 0.03	0.30 ± 0.06
Proposed (LD)	0.55 ± 0.01	0.48 ± 0.01	**0.34 ± 0.04**	0.36 ± 0.05
Proposed(gene)	0.56 ± 0.02	0.47 ± 0.01	0.22 ± 0.02	**0.39 ± 0.03**
Proposed(tagSNP)	0.60 ± 0.03	0.52 ± 0.01	0.26 ± 0.05	0.27 ± 0.04

*The best correlation coefficients are shown in boldface.*

### The Top Selected ROIs

**FIGURE 5:**
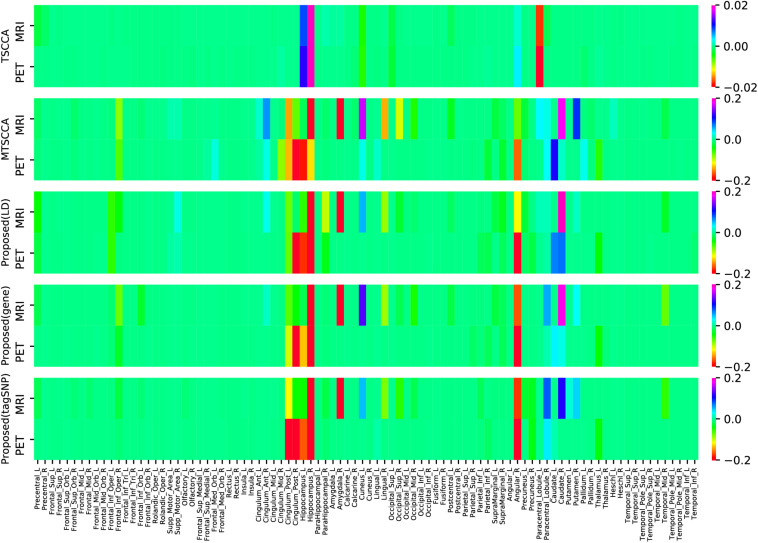
Comparison of estimated canonical weights of imaging QTs. Each row represents: (1) TSCCA; (2) MTSCCA; (3) Proposed (LD); (4) Proposed (gene); (5) Proposed (tagSNP). Within each row, there are two parts represent two imaging modalities.

In addition to the CCCs, the canonical weights were also one of the focuses of our study since they can help us find brain regions being highly related to AD. Figure 5 shows the comparison of mean canonical weights of two imaging QTs based on five-fold cross-validation trials. Each row represents an SCCA model. The heatmap color represents the estimated weight of each model, so the selected QTs were highlighted in Figure 5. We can clearly observe that several brain regions were selected by both MRI and PET scans, such as the right hippocampal and the right angular gyrus, indicating that these regions may be modality-consistent. Additionally, TSCCA identified only modality-consistent QTs but failed to identify modality-specific QTs. This was due to the nature of its modeling strategy and may have resulted in crucial biomarkers being ignored. Multi-task models can identify modality-specific and modality-consistent QTs, which also implied the limitations of conventional multi-view SCCA models. In order to more accurately analyze the identified brain regions, using the proposed model with LD group information, the top ten ROIs of each modality were selected and sorted according to the absolute values of canonical weights.

As shown in Table 4, ROIs that were jointly selected by two imaging modalities are shown in boldface, all of which are known to be closely related to the pathogenesis of AD according to previous research. The hippocampus is essential for forming new memories and was reported as one of the earliest affected brain regions in AD and MCI (Moreno-Jimenez et al., 2019). Both left and right caudate nucleus have been reported that their volume is significantly different between AD and normal control (Cho et al., 2014; Botzung et al., 2019). The right angular gyrus is considered to be closely related to language ability, and patients with angular gyrus syndrome are often found to have damage in this brain area (Horwitz et al., 1998). The right parahippocampal gyrus affects the encoding and maintenance of bound information related to working memory (Luck et al., 2010). The metabolic reduction in the posterior cingulate gyrus is a very early sign in AD (Minoshima et al., 1997). Notably, all the remaining brain regions have also been reported to be associated with AD in published literature. These satisfactory results were due to the inclusion of cognitive information into the linear regression to adjust weighting.

**TABLE 4:** The top ten selected ROIs by the proposed model.

MRI	PET
**Hippocampus_R**	Cingulum_Post_R
Amygdala_R	**Angular_R**
**Caudate_R**	**Hippocampus_R**
**Angular_R**	**Hippocampus_L**
**ParaHippocampal_R**	**Caudate_R**
Lingual_R	Caudate_L
**Cingulum_Post_L**	**Cingulum_Post_L**
Cuneus_L	**Frontal_Inf_Oper_L**
**Hippocampus_L**	Thalamus_L
**Frontal_Inf_Oper_L**	**ParaHippocampal_R**

*The jointly selected ROIs are shown in boldface.*

**FIGURE 6:**
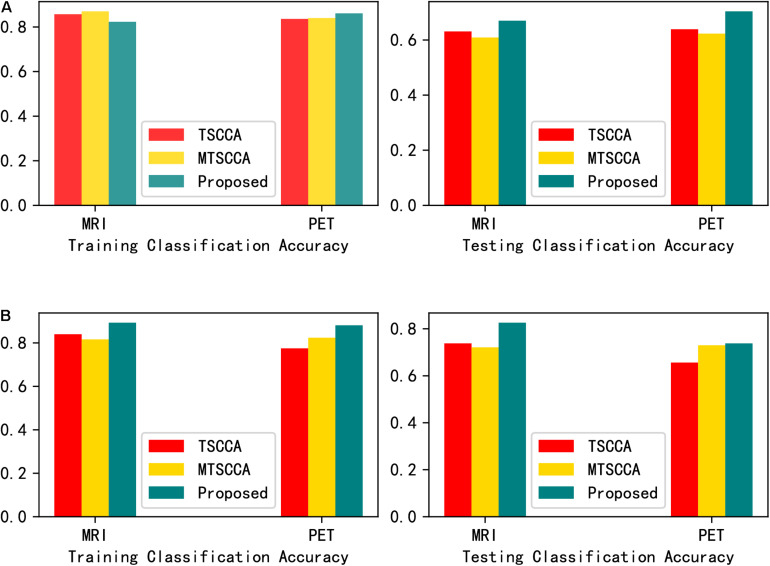
**(A)** Comparison of the classification accuracy of the selected imaging QTs by support vector machine (SVM). **(B)** Comparison of the classification accuracy of the selected imaging QTs by random forest (RF).

In order to further thoroughly verify that the neuroimaging biomarkers found by the proposed model are more disease-related than those found by the other two models. Selecting the top ten QTs as input features, support vector machine (SVM) with Gaussian radial basis function (RBF) kernel and random forest (RF) were adopted as classification methods. The parameters were tuned with five-fold cross-validation based on the training sets. Figure 6 presents the classification accuracies of the two classifiers. The testing classification results showed that the classifier using the features selected by MT-SCCAR achieved the highest accuracies, thus indicating the superiority of MT-SCCAR in identifying disease-related biomarkers. Notably, the testing classification accuracies were relatively low for both SVM and RF, probably due to inevitable noise during the feature extraction process of brain imaging. These results were also consistent with previous studies (Wang et al., 2012b; Adeli et al., 2017).

### The Top Selected SNPs

In addition to neuroimaging biomarkers, SCCA models can also identify genetic biomarkers. We averaged the SNP canonical weights into a single vector and selected the top ten SNPs. As illustrated in Table 5, the proposed model with LD or gene group information yielded meaningful results. For example, rs769449 (APOE) is located in promoter and enhancer areas for multiple brain tissues and is associated with AD (Liu et al., 2018). Moreover, the well-known AD risk biomarker rs429358 (APOE) was also identified by the proposed model, demonstrating its strong correlation ability. The remaining five SNPs of the proposed model, i.e., rs7256200 (3.3 kb of APOE), rs10414043 (3kb of APOE), rs4901317 (FERMT2), rs449647 (0.5 kb of APOE), and rs405509 (0.2 kb of APOE), have also been documented to increase the risk of AD in previous studies (Lin et al., 2017; Xiao et al., 2017). However, four selected SNPs have not yet been reported to be related to AD. They still need further research to confirm in the future. Next, we compared the top ten SNPs identified by MT-SCCAR (LD and gene) with the 894 tagSNPs. Interestingly, MT-SCCAR (LD) identified six tagSNPs (rs7256200, rs4901317, rs429358, rs7157639, rs449647, and rs3829947). Multi-task sparse canonical correlation analysis and regression (gene) identified five tagSNPs (rs7256200, rs7157639, rs405509, rs429358, and rs4901317). This implied that using tagSNP will reduce the number of SNPs that need to be analyzed and facilitate identifying significant SNPs. The proposed model with tagSNP also identified some significant SNPs. For example, rs59325138 (3.6 kb of APOE) has been reported to modify the cerebrospinal fluid apolipoprotein E protein levels (Cervantes et al., 2011). The Beta-Amyloid (1-42), an AD biomarker, is associated with rs439401 (1.8kb of APOE) (Xu et al., 2014). The TSCCA identified the rs4292358 and three other SNPs (rs405509, rs7412, and rs7794735) that have been reported previously (Arking et al., 2008; Ma et al., 2016; Zhen et al., 2017). The MTSCCA also identified four SNPs (rs769449, rs7256200, rs10414043, and rs7794735) but cannot identify rs429358. In summary, the proposed model was more accurate for identifying disease-specific genetic biomarkers than the other two models.

**TABLE 5:** The top ten selected SNPs.

TSCCA	MTSCCA	Proposed (LD)	Proposed (gene)	Proposed (tagSNP)
rs735780	rs769449	rs769449	rs7256200	rs117641527
rs405509	rs7256200	rs7256200	rs10414043	rs8012948
rs578506	rs10414043	rs10414043	rs769449	rs1884910
rs4904901	rs4904901	rs4901317	rs7157639	rs78015388
rs7157639	rs61975596	rs429358	rs405509	rs2598123
rs429358	rs7794735	rs4904901	rs4904901	rs4335936
rs4257390	rs55636820	rs7157639	rs429358	rs59325138
rs7412	rs77640937	rs449647	rs75773078	rs439401
rs7794735	rs34273097	rs11629428	rs11629428	rs112097633
rs10256195	rs9972149	rs3829947	rs4901317	rs429358

**FIGURE 7:**
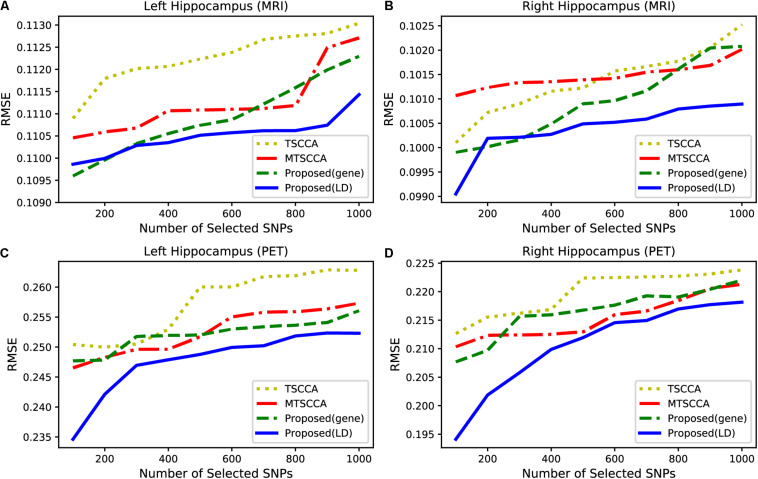
Comparison of the RMSE with respect to different numbers of SNPs from 100 to 1000. **(A)** QT of left hippocampus based on MRI scan. **(B)** QT of right hippocampus based on MRI scan. **(C)** QT of left hippocampus based on PET scan. **(D)** QT of right hippocampus based on PET scan.

Alzheimer’s disease (AD) usually first affects the hippocampus, resulting in cognitive decline and memory loss (Moreno-Jimenez et al., 2019). Therefore, when selecting the same number of features, the predictive effect of the QTs of the hippocampus can be used to evaluate model performance. Based on this analysis, we built a regression model to predict the QTs of the hippocampus from MRI and PET scans. Different numbers of SNPs were selected from 100 to 1000 with a step of 100. Using a support vector machine (SVR) with RBF kernel, we calculated the average root mean squared error (RMSE) for each model based on five-fold cross-validation. For a fair comparison, we only compared TSCCA, MTSCCA, MT-SCCAR (gene), and MT-SCCAR(LD) since MT-SCCAR (tagSNP) used only 894 tagSNPs. Figure 7 shows the testing RMSE of the left and right hippocampus obtained by different imaging techniques. Smaller RMSE indicates that the selected SNPs are more related to AD. According to Figure 7, the prediction errors were lowest for the proposed model. These results suggested that the proposed model outperformed the other two models on four imaging QTs.

### Pairwise Correlation Analyses

**FIGURE 8:**
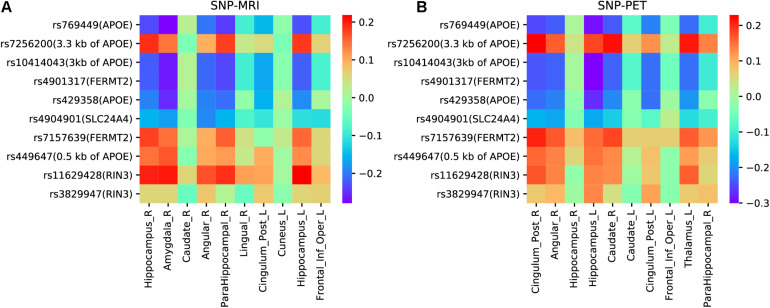
**(A)** The heatmap of pairwise correlations between SNPs and ROIs of MRI scan. **(B)** The heatmap of pairwise correlations between SNPs and ROIs of PET scan.

Based on the top ten selected ROIs and SNPs obtained by the proposed model with LD group information, we drew heatmaps of pairwise correlation coefficients between SNPs and two imaging QTs. As illustrated in Figure 8, it is clearly observed that the selected SNPs were mainly located in and around the APOE region. APOE is the major genetic risk factor for AD (Munoz et al., 2019). Moreover, the association patterns of SNPs and ROIs selected by MRI and PET were very similar, which indicated the ability of our model to identify modality-consistent biomarkers.

To gain more insight, we further analyzed four undocumented SNPs (rs4904901, rs7157639, rs11629428, and rs3829947) identified by MT-SCCAR with LD group information. The imaging QTs which had the strongest association with these four SNPs were singled out. Consequently, a total of eight SNP-ROI pairs were generated to validate the proposed model. These associations can also allow us to explore relationships from the microscopic molecular level to the macroscopic brain level. Table 6 shows the Pearson correlation coefficients and p-values of eight SNP-ROI pairs. The p-values of all eight pairs were small, indicating a significant correlation within each pair. For rs4904901,it was correlated strongest with the same brain region across both imaging modalities, which suggests it is a modality-consistent association pattern. For the rest of the SNPs, the heterogeneous association patterns may have great potential to help us understand how changes in molecular level influence brain structure and metabolic.

**TABLE 6:** The correlation coefficients and p-values of eight SNP-ROI pairs.

SNP-ROI pairs	Correlation coefficient	p-value
rs4904901-Angular_R(MRI)	−0.189	0.002
rs4904901- Angular_R(PET)	0.180	0.003
rs7157639-Hippocampus_R(MRI)	0.176	0.003
rs7157639-Cingulum_Post_R(PET)	0.204	0.001
rs11629428-Hippocampus_L(MRI)	0.218	0.0003
rs11629428- Cingulum_Post_R(PET)	0.171	0.004
rs3829947- Angular_R(MRI)	0.078	0.067
rs3829947- Hippocampus_L(PET)	0.135	0.025

## Conclusion

In this paper, we proposed the MT-SCCAR model to investigate potential neuroimaging and genetic biomarkers. Compared with TSCCA and MTSCCA, the proposed model integrated genotype, multiple neuroimaging, and neuropsychological assessments into a single model to analyze multi-modal information. We tested our model on synthetic and ADNI data sets and compared its association results with those of TSCCA and MTSCCA. We found that our model demonstrated higher CCCs of 0.34 ± 0.04 (LD) and 0.39 ± 0.03 (gene) compared with the CCCs of TSCCA (0.23 ± 0.03) and MTSCCA (0.30 ± 0.06). Moreover, MT-SCCAR identified a small number of SNPs from enormous SNPs that were related to AD, wherein all of the top ten selected ROIs were AD brain risk regions. These satisfactory results show that MT-SCCAR outperforms TSCCA and MT-SCCA in detecting disease-specific biomarkers on multi-modal data.

The proposed model incorporates SNPs, neuroimaging measurements, and cognitive scores. However, there are a number of biological pathways that correlate with structural changes in the brain. Therefore, future efforts should aim to integrate data across more levels (i.e., gene expression, cell, and DNA methylation) for a more sophisticated understanding of the biological pathways leading from gene to disease.

## Data Availability Statement

The datasets for this article are not publicly available but are available upon request at the following private repository: Alzheimer’s Disease Neuroimaging Initiative, http://adni.loni.usc.edu/data-samples/access-data/, https://ida.loni.usc.edu/pages/access/studyData.jsp (The dataset contains the neuropsychological assessment data), and https://ida.loni.usc.edu/pages/access/geneticData.jsp (The dataset contains the genetics data). Requests to access the datasets should be directed to (Alzheimer’s Disease Neuroimaging Initiative or catherine.conti@ucsf.edu). The code is available at https://github.com/ftorange/MT-SCCAR.

## Author Contributions

WK and FK designed the model and analyzed the results. FK prepared data and drafted the manuscript. SW and FK performed the pre-processing with imaging and genetics data. WK helped with data interpretation and manuscript drafting. All authors read and approved the final manuscript.

## Conflict of Interest

The authors declare that the research was conducted in the absence of any commercial or financial relationships that could be construed as a potential conflict of interest.

## Publisher’s Note

All claims expressed in this article are solely those of the authors and do not necessarily represent those of their affiliated organizations, or those of the publisher, the editors and the reviewers. Any product that may be evaluated in this article, or claim that may be made by its manufacturer, is not guaranteed or endorsed by the publisher.

## Footnotes

1
http://adni.loni.usc.edu/

2
www.alzgene.org
